# Recent Advances in Conjugated Polymer-Based Microwave Absorbing Materials

**DOI:** 10.3390/polym9010029

**Published:** 2017-01-14

**Authors:** Ying Wang, Yunchen Du, Ping Xu, Rong Qiang, Xijiang Han

**Affiliations:** MIIT Key Laboratory of Critical Materials Technology for New Energy Conversion and Storage, School of Chemistry and Chemical Engineering, Harbin Institute of Technology, Harbin 150001, China; wangying901115@163.com (Y.W.); pxu@hit.edu.cn (P.X.); qiangrong2009@126.com (R.Q.)

**Keywords:** conjugated polymers, microwave absorbing materials, microstructure, composites, reflection loss

## Abstract

Microwave absorbing materials (MAMs) are paving the way for exciting applications in electromagnetic (EM) pollution precaution and national defense security, as they offer an advanced alternative to conventional reflection principles to fundamentally eliminate the EM waves. Conjugated polymer (CP)-based composites appear as a promising kind of MAM with the desirable features of low density and high performance. In this review, we introduce the theory of microwave absorption and summarize recent advances in the fabrication of CP-based MAMs, including rational design of the microstructure of pure conjugated polymers and tunable chemical integration with magnetic ferrites, magnetic metals, transition metal oxides, and carbon materials. The key point of enhancing microwave absorption in CP-based MAMs is to regulate their EM properties, improve matching of characteristic impedance, and create diversified loss mechanisms. The examples presented in this review will provide new insights into the design and preparation of CP-based composites that can satisfy the high demands of the oncoming generation of MAMs.

## 1. Introduction

The extensive utilization of communication devices—e.g., telecommunications, local area network systems, and radar systems—currently generates a large amount of electromagnetic (EM) waves into the living space of human beings. This serious EM emission leads to grim problem of EM interference (EMI), which not only causes damage to highly sensitive electronic equipment, but also has a remarkable negative effect on physical health [1,2,3,4]. To resolve the issues associated with EMI, numerous efforts have been made on EMI shielding referring to the essential blockage of EM radiation so that it cannot pass through the shielding layer [5]. However, the shielding materials are actually unable to dissipate EM emission due to their reflection principle for incident EM waves, and worse than that, they also produce secondary/repeated EMI pollution. In recent years, microwave-absorbing materials (MAMs) with low reflection and high absorption have received increasing attention due to their pragmatic and effective functions for reducing EMI pollution, because they can intrinsically convert the EM energy into thermal energy or dissipate EM waves through destructive interference [6,7].

To satisfy the requirements of practical applications, an eligible absorber should be labeled with some characteristic features, including being lightweight and thin, with powerful absorption and wide frequency bandwidth [8,9]. Generally, MAMs can be classified into two categories on the basis of their microwave loss mechanism: dielectric loss materials and magnetic loss materials [10]. Magnetic loss materials such as Fe [11], Co [12], Ni [13], and related ferrites [14,15] normally possess good microwave-absorbing performances in strong reflection loss and wide frequency response, while their high density and poor corrosion resistance restrict their sustainable development. In contrast, traditional dielectric loss materials such as SiC [16], BaTiO_3_ [17], carbon [18], and ZnO [19] also frequently suffer from mismatched impedance and narrower absorption bandwidth resulting from the relatively large gap between their relative complex permittivity and relative complex permeability. Therefore, developing novel MAMs with excellent performance is still a challenging task in the field of microwave absorption.

Since the discovery of conjugated polymers (CPs), many potential applications have been explored, ranging from organic electronic devices (such as polymer light-emitting diodes (PLEDs) [20,21], polymer solar cells (PSCs) [22,23], organic field effect effect transistors (OFETs) [24,25], and chemo-/bio-sensors [26,27]. Different from traditional polymers, the π-conjugated main chains in CPs give them a delocalized electronic structure which endows them with unusual electronic properties, such as electrical conductivity, low energy optical transitions, low ionization potential, and high electron affinity [28]. The high electrical conductivity gifted by the extended π-conjugated system even gains them the name of “synthetic metal”. As a result, they have been widely studied as EM shielding materials in earlier studies [29,30,31]. However, it was also found that the shielding effectiveness of CPs came partially from their available microwave absorption performance rather than just from reflection [31]. This valid absorption performance motivated a great deal of research work on developing novel MAMs based on various CPs. In addition, the unique advantages of CPs (e.g., low density, low cost, resistance to corrosion, and ease of preparation [32]) also render them as one kind of the most promising candidates for MAMs in practical applications. To date, CP-based MAMs have made significant achievements in the field of microwave absorption through rational design on microstructure and hybridization with different types of materials [33,34]. With more and more publications in this field, a comprehensive summary and review of the published research results and relevant theory will be very helpful to further promote the development of the microwave absorption of CP-based MAMs. Therefore, in this review, we not only summarize theoretical calculations, loss mechanisms, and evaluation methods, but also highlight the recent advances of CP-based MAMs, including pure CPs, magnetic ferrites/CP composites, magnetic metal/CP composites, transition metal oxides/CP composites, carbon/CP composites, multi-compound CP-based composites, and multi-layer CP-based composites. Furthermore, the shortcomings, challenges, and prospects of CP-based MAMs are also discussed.

## 2. Theory of Microwave Absorption

**Figure 1:**
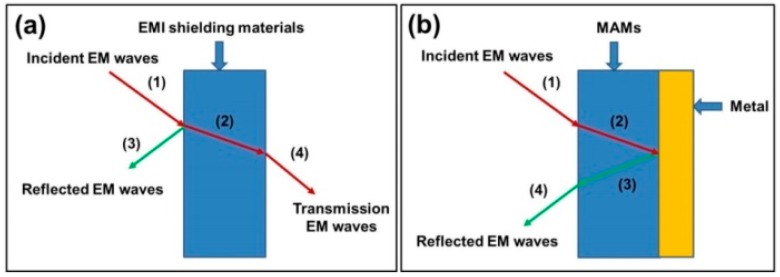
The evaluation models of (**a**) conventional electromagnetic interference (EMI) shielding and (**b**) microwave absorption. MAM: microwave-absorbing material.

EMI shielding and microwave absorption are two common strategies to resist the interference of incident EM waves, while they are generally evaluated by different measurement models because of their distinguishable concerns. For a conventional EMI shielding model (Figure 1a), the most important thing is to attenuate the intensity of the transmission EM waves, and a common parameter—shielding effectiveness (SE)—is employed to describe the relationship between the transmission wave and the incident wave (wave (1) and wave (4) in Figure 1a), which can be defined as

SE = *R* + *A* + *B* (1)

where *R* is the reflection loss, *A* is the absorption loss, and *B* stands for the multiple reflection loss [35]. In this case, high reflectivity is very favorable to the SE, and most incident waves are reflected off the surface of shielding materials. The physical significance of reflection loss in EMI shielding is the difference between the initial incident waves and those waves penetrating into the shielding materials (wave (1) and wave (2) in Figure 1a). For the model of microwave absorption (Figure 1b), however, a metal substrate is placed to reflect the transmission waves. As a result, the transmission waves are always negligible in microwave absorption. Reflection loss herein means the difference between the initial incident waves and the final reflected waves (wave (1) and wave (4) in Figure 1b). The final reflected waves include all back-propagation EM waves reflected at various surfaces and interfaces, and ideal MAMs should weaken the reflected waves as much as possible. Relative complex permittivity (ε_r_ = ε′ − *j*ε″) and relative complex permeability (μ_r_ = μ′ − *j*μ″) are very important parameters that can determine the microwave absorption properties of an absorber. The real parts of relative complex permittivity (ε′) and relative complex permeability (μ′) are on behalf of the storage capability of electric and magnetic energy, and the imaginary parts (ε″ and μ″) describe the loss capability of electric and magnetic energy [36]. On the basis of relative complex permittivity and relative complex permeability, the reflection loss (*R*_L_(dB)) properties of an absorber can be deduced from the transmission line theory [37]:

(2) $$R_{L}\left(\mathrm{dB}\right)=20\log|\frac{Z_{\mathrm{in}}-1}{Z_{\mathrm{in}}+1}|$$

**Figure 2:**
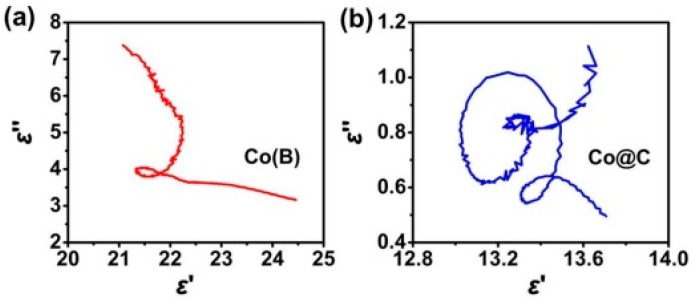
Plots of ε″ versus ε′ for (**a**) metallic Co and (**b**) core–shell Co@C microspheres. Reprinted with permission from Reference [46].

*Z*_in_ refers to the normalized input impedance of a metal-backed microwave absorbing layer, and is given by

(3) $$Z_{\mathrm{in}}=\sqrt{\frac{\mu_{r}}{\varepsilon_{r}}}\tanh\left[j\left(\frac{2\pi }{c}\right)fd\sqrt{\mu_{r}\varepsilon_{r}}\right]\;$$

where *c* is the velocity of EM waves in free space, *f* is the microwave frequency, and *d* is the thickness of an absorber. It is widely accepted that dielectric loss ability mainly comes from conductivity loss and polarization loss [38,39], and the polarization loss can be further divided into ionic polarization, electronic polarization, dipole orientation polarization, and interfacial polarization (space charge polarization) [40]. According to the free electron theory [41], $\varepsilon^{\prime \prime }\approx 1/2\pi \rho f\varepsilon_{0}$, where *ρ*, ƒ, and $\varepsilon_{0}$ are the resistivity, the frequency, and the dielectric constant of free space, respectively, it can be expected that high electric conductivity (i.e., low resistivity) will enhance the imaginary parts of relative complex permittivity. Ionic polarization and electronic polarization can be easily excluded in microwave absorption because they usually occur at a much higher frequency region (10^3^–10^6^ GHz) [40]. Dipoles—namely, bound charges in dielectric medium—are generally restricted on the defects and residual groups, and cannot move freely like electrons in an external electric field. Under a high-frequency alternating electric field, the dipoles cannot reorient themselves quickly enough to respond to the applied electric field, resulting in dipole orientation polarization [42]. At the same time, ε′ and ε″ will start to decrease and produce typical frequency dispersion behaviors [42]. The contribution from interfacial polarization always appears in a heterogeneous system, and the accumulation and uneven distribution of space charges at the interfaces will produce a macroscopic electric moment that can consume the incident EM energy effectively [43,44]. To better understand the polarization relaxations, Cole and Cole reinterpreted the classical Debye equations into [45]

(4) $${\left(\varepsilon^{\prime }-\varepsilon_{\infty }\right)}^{2}+{\varepsilon^{\prime \prime }}^{2}={\left(\varepsilon_{s}-\varepsilon_{\infty }\right)}^{2}$$

where $\varepsilon_{s}$ and $\varepsilon_{\infty }$ are the static dielectric constant and the dielectric constant at infinite frequency, respectively. If there is a polarization relaxation process, the plot of ε″ versus ε′ will be a single semicircle, which is also defined as the Cole–Cole semicircle. To better illustrate the application of the Cole–Cole semicircle, Figure 2 displays the plots of ε″ versus ε′ of metallic Co and core–shell Co@C microspheres in wax matrix [46]. As observed, metallic Co gives only one semicircle due to the interfacial polarization relaxation between Co and wax, while Co@C microspheres display at least three semicircles in the studied frequency range. The additional semicircles in Co@C microspheres mean more relaxations from multiple interfacial polarizations and dipole orientation polarization.

Magnetic loss usually becomes active in the presence of magnetic components in MAMs, and it mainly originates from hysteresis, domain wall resonance, natural ferromagnetic resonance, and eddy current effect [47]. However, natural ferromagnetic resonance and eddy current effect are taken as two dominant factors that contribute to magnetic loss, because the hysteresis loss is negligible in the weak EM field and domain wall resonance only occurs at much lower frequency (1–100 MHz) [47]. According to the ferromagnetic resonance theory [48], the natural ferromagnetic resonance frequency correlates to an anisotropy field which can be expressed by the equation

(5) $$f_{r}=\left(\gamma /2\pi \right)H_{\mathrm{eff}}$$

where $f_{r}$ is the resonance frequency, γ is the gyromagnetic ratio, and $H_{\mathrm{eff}}$ is the effective anisotropy field. As the smaller magnetic particles can lead to an enhanced effective anisotropy, the ferromagnetic resonance in high-frequency range can be expected by decreasing the size of the magnetic particle [49]. The eddy current loss is universal in ferromagnetic conductors and it can be expressed by [50]:

(6) $$\mu^{\prime \prime }=2{\pi \mu }_{0}{\left(\mu^{\prime }\right)}^{2}\sigma \cdot d^{2}f/3$$

where σ (S·m^−1^) is the electrical conductivity and $\mu_{0}$ (H·m^−1^) is the permeability in vacuum. If the microwave attenuation only results from the eddy current effect, the values of μ″ (μ′)^−2^*f*^−1^ will be constant under varied frequency [47,49]. It is worth noting that the particle size of the ferromagnetic conductor always plays an important role in determining the eddy current effect. Once the particle size exceeds the critical value, a strong eddy current will generate a skin effect, resulting in the partial invalidation of the internal magnetic field and consequent degradation of relative complex permeability. Therefore, decreasing the particle size of the ferromagnetic conductor and constructing multi-component composites are two familiar strategies to suppress the skin effect [51,52]. Although some common magnetic parameters—such as saturation magnetization (*M*_S_) and coercivity (*H*_C_)—cannot be intuitively linked with magnetic loss, another important concept, initial permeability (μ_i_), is usually employed to predict magnetic loss ability [13,53], and can be expressed by

(7) $$\mu_{i}=\frac{M_{s}^{2}}{akH_{c}M_{s}+b\lambda \zeta }$$

where *a* and *b* are two constants determined by the material composition, *λ* is the magnetostriction constant, and ξ is an elastic strain parameter of the crystal. For ferromagnetic MAMs, high μ_i_ will favor strong magnetic loss ability, and it can be concluded from Equation (7) that larger *M*_S_ and smaller *H*_C_ may contribute to the improvement of μ_i_ value.

For direct evaluation of the dielectric loss and magnetic loss abilities, two common concepts—dielectric dissipation factor (tan*δ*_e_ = ε″/ε′) and magnetic dissipation factor (tan*δ*_m_ = μ″/μ′)—are widely utilized. Although a large dielectric dissipation factor and magnetic dissipation factor are desirable in MAMs, the specific microwave absorption does not simply depend on dielectric loss ability or magnetic loss ability. There is another important parameter—the concept of matched characteristic impedance, relating to the reflection loss properties of various MAMs—that can determine the transmission behaviour of incident EM waves [54]. In theory, the characteristic impedance of MAMs should be equal/close to that of the free space (377 Ω) to achieve zero-reflection at the front surface of the materials [54]. If the characteristic impedance is well matched, most EM waves can enter into MAMs to be converted into thermal energy or dissipated through interference; otherwise, most EM waves will be reflected at the front surface of MAMs or pass through the MAMs without any dissipation [55,56]. Recently, Ma et al. proposed a delta-function method to validate the impedance matching degree of various absorbers by the following equation [57]:

(8) $$\left|\Delta \right|=\left|\sinh^{2}\left(Kfd\right)-M\right|$$

where |∆| represents the impedance mismatch degree, and *K* and *M* are determined by the relative complex permittivity and permeability

(9) $$K=\frac{4\pi \sqrt{\mu^{\prime }\varepsilon^{\prime }}\cdot \sin\frac{\delta_{e}+\delta_{m}}{2}}{c\cdot \cos\delta_{e}\cos\delta_{m}}$$

(10) $$M=\frac{4\mu^{\prime }\cos\delta_{e}\varepsilon^{\prime }\cos\delta_{m}}{{\left(\mu^{\prime }\cos\delta_{e}-\varepsilon^{\prime }\cos\delta_{m}\right)}^{2}+{\left[\tan\left(\frac{\delta_{m}}{2}-\frac{\delta_{e}}{2}\right)\right]}^{2}{\left(\mu^{\prime }\cos\delta_{e}+\varepsilon^{\prime }\cos\delta_{m}\right)}^{2}}$$

Therefore, higher absorption at a thickness is the result of better impedance match, where |Δ| is close to zero. If characteristic impedance of MAMs is not satisfying the criteria, |Δ| tends to stay away from zero, giving poor microwave absorption.

## 3. Pure CPs as MAMs

**Figure 3:**
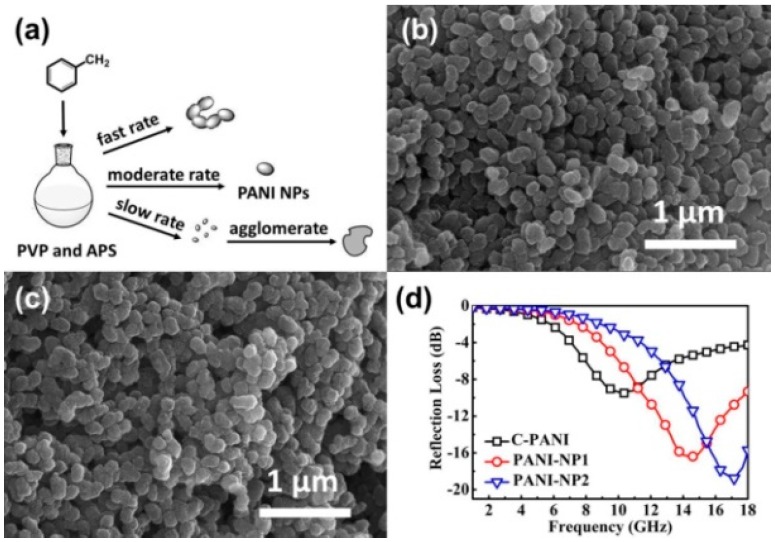
(**a**) Schematic illustration of the possible mechanism for polyaniline (PANI) nanoparticles (NPs); SEM images of (**b**) PANI-NP1 and (**c**) PANI-NP2; (**d**) Reflection-loss curves of conventional PANI (C-PANI), PANI-NP1, and PANI-NP2 with an absorber thickness of 2 mm in the frequency range of 1–18 GHz. Reprinted with permission from Reference [62]. APS: ammonium persulfate; PVP: Polyvinylpyrrolidone.

Although the microwave absorption ability of CPs has been recognized in earlier study, the performance of pure CPs is not that exciting, because their relatively high relative complex permittivity and quite low relative complex permeability are not favorable for the matching of characteristic impedance, resulting in serious deviation from the zero-reflection condition at the surface of MAMs [54]. Chandrasekhar et al. investigated the microwave shielding and absorption of bulk polyaniline (PANI) doped with two proprietary sulfonate dopants, and they found that bulk PANI has good shielding effectiveness (<−15 dB over 4 to 18 GHz) and poor absorption performance (about −5 dB in X and K bands) [31]. Very interestingly, recent progress indicates that rational design on the microstructure of CPs will be an effective strategy to regulate the EM properties, improve the matching of characteristic impedance, and enhance the microwave absorption. For example, Zhang et al. prepared PANI nanofibers and nanoparticles by interfacial polymerization method, and the resultant PANI nanofibers exhibited uncommon negative relative complex permittivity and dielectric dissipation factor [58]. Sun et al. reported that PANI microrods could show stronger reflection loss than conventional PANI [59]. Xie et al. designed an ultralight three-dimensional (3D) polypyrrole (PPy) aerogel and (3D) polypyrrole/poly(3,4-ethylenedioxythioxythiophene) (PPy/PEDOT) hybrid composite by a facile self-assembled polymerization method, and the effective EM absorption bandwidth (<−10 dB, 90% absorption) could reach up to 6.2 GHz [60,61]. Our group previously performed the synthesis of PANI nanoparticles using a reverse dropping method with PVP (Polyvinylpyrrolidone) as a steric stabilizer [62]. The morphology of PANI was highly dependent on the dropping rate (Figure 3a), and well-dispersed PANI nanoparticles could only be obtained under optimum conditions (Figure 3b,c). The different dropping rate also led to significant changes in the length and oxidation-state of conjugated chains, and thus the resultant products presented quite distinguishable EM properties and microwave absorption characteristics. Especially, PANI-NP1 and PANI-NP2—consisting of well-dispersed nanoparticles—displayed substantially enhanced reflection loss as compared with conventional PANI (C-PANI) (Figure 3d).

**Figure 4:**
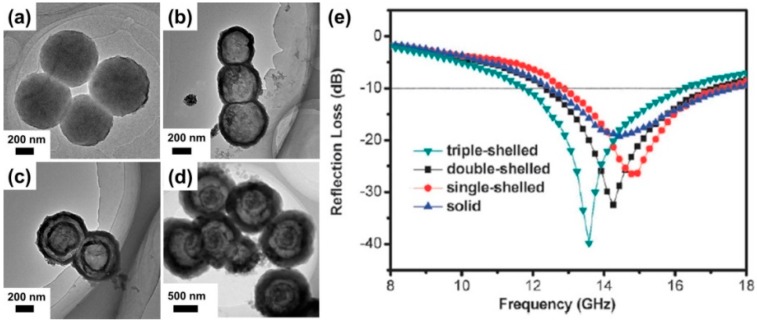
(**a**) TEM images of poly(3,4-ethylenedioxythioxythiophene (PEDOT) solid spheres; (**b**) single-shelled PEDOT hollow spheres; (**c**) double-shelled PEDOT microspheres; and (**d**) triple-shelled PEDOT microspheres; (**e**) Reflection loss for different morphologies of PEDOT microspheres in the frequency range of 8.0–18.0 GHz with a thickness of 2 mm. Reprinted with permission from Reference [34].

According to the transmission line theory, multiple reflections or scattering inside absorbers can promote the attenuation of incident EM waves [35]. Therefore, it is of great significance to create some unique microstructure (e.g., hollow, core–shell, yolk–shell) in CPs, which can facilitate multiple reflections/scatterings to further enhance microwave absorption. As shown in Figure 4a–d, multi-shelled PEDOT hollow microspheres were generated by using Fe_3_O_4_ hollow microspheres as sacrificial templates via a programmed reaction-temperature process [34]. It could be found that with an increase in the number of shells, the microwave absorption properties of PEDOT microspheres were obviously enhanced (Figure 4e). The maximum *R*_L_ values of the triple-shelled and double-shelled PEDOT were 39.7 and 32.4 dB, respectively, at a thickness of 2 mm, which are higher than those of PEDOT solid particles (19.2 dB) and single-shelled PEDOT (26.5 dB). The excellent microwave absorption performance of the multiple-shelled PEDOT microspheres was attributed to the following aspects: (1) the existing interfacial polarization between multiple core/shell/shell gradient interfaces were extremely favorable for EM attenuation; (2) high void space in these multiple shell microspheres provided more active sites for the reflection and scattering of incident EM waves than the solid PEDOT microspheres. By considering the importance of interfacial polarization, our group also constructed highly uniform core–shell PPy@PANI composites with controllable PANI shell thickness [34]. The as-prepared composites showed much better microwave absorption than individual PPy microspheres and PANI. Investigations on the mechanism indicated that interfacial polarization contributed to the dielectric loss significantly, which was beneficial for the creation of well-matched characteristic impedance in these core–shell composites and producing strong reflection loss.

## 4. CP-Based Composites as MAMs

Compared with the design of CP microstructure, there have been more tremendous interests in constructing CP-based heterogeneous nanocomposites for microwave absorption applications, because the embedment of heterogeneous components into the CP matrix is a relatively simple approach to manipulating the EM properties and creating additional loss mechanisms. To date, various magnetic ferrites, magnetic metals, transition metal oxides, and diversified carbon materials have been extensively coupled with CPs to produce novel MAMs with better performance.

### 4.1. Magnetic Ferrites/CP Composites

**Figure 5:**
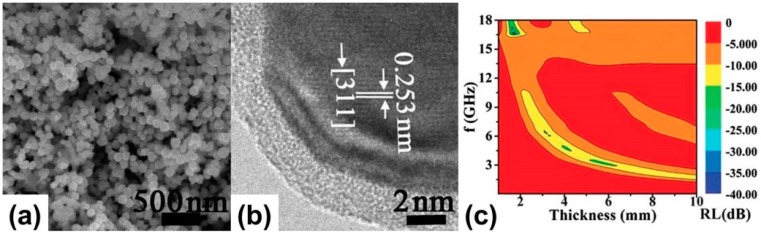
(**a**) SEM image; (**b**) HRTEM (High Resolution Transmission Electron Microscopy) image; and (**c**) two-dimensional (2D) representation of the *R*_L_ of Fe_3_O_4_–PANI nanoparticles. Reprinted with permission from Reference [78].

As a kind of typical magnetic material, spinel ferrites (e.g., Fe_3_O_4_, γ-Fe_2_O_3_, CoFe_2_O_4_, NiFe_2_O_4_, Mn_1-*x*_Zn*_x_*Fe_2_O_4_) have been taken as the most common additives in CP-based MAMs due to their simple chemical composition and mild preparation [63,64,65,66,67,68,69,70,71,72,73,74,75,76,77]. For example, Yang et al. decorated hollow PANI microspheres with well-dispersed Fe_3_O_4_ nanoparticles, and they found that the resultant composites could produce much better reflection loss characteristics than sole PANI microspheres [66]. Gandhi et al. synthesized conducting ferromagnetic polyaniline-CoFe_2_O_4_ nanocomposites via the one-step chemical oxidative polymerization of aniline in the presence of CoFe_2_O_4_ nanoparticles (30–40 nm), and the incorporation of CoFe_2_O_4_ nanoparticles was confirmed to be responsible for the enhanced absorption effectiveness in the frequency range of 12.4–18.0 GHz (Ku band) [70]. Li et al. coated flake-like NiFe_2_O_4_ with PANI and PPy, respectively, and EM analysis revealed that the NiFe_2_O_4_/PPy composite would be an eligible candidate for microwave absorption [77]. Although an expected improvement in properties can be achieved by these composites, it is important to mention that spinel ferrite particles are usually randomly distributed in the matrix of CPs, and the poor chemical homogeneity will not be favorable for their practical applications [32,55]. As a result, some uniform core–shell composites were elaborately developed to remedy the drawbacks in conventional composites [78,79]. As observed in Figure 5, the uniformly continuous PANI coating on the surface of Fe_3_O_4_ nanoparticles provided excellent chemical homogeneity, and the synergistic effect between PANI coating and Fe_3_O_4_ nanoparticles could guarantee the enhanced microwave absorption performance as compared with independent PANI and Fe_3_O_4_ [78].

**Figure 6:**
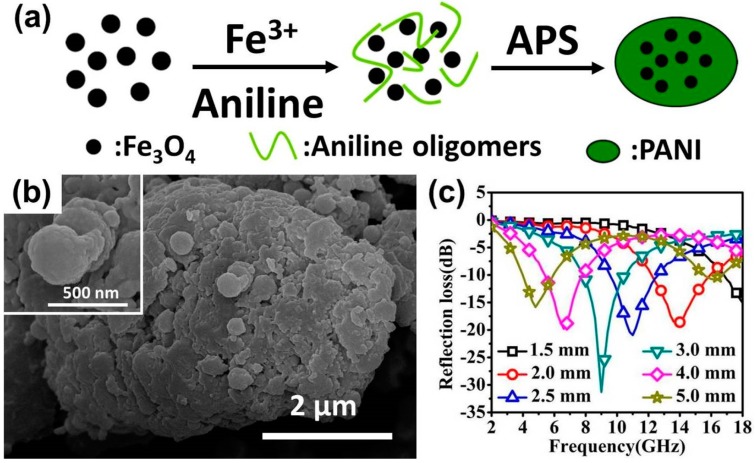
(**a**) Possible formation mechanism; (**b**) SEM image; and (**c**) reflection loss curves dependent on the thickness of “egg-like” Fe_3_O_4_ microspheres/PANI composites. Reprinted with permission from Reference [86].

**Figure 7:**
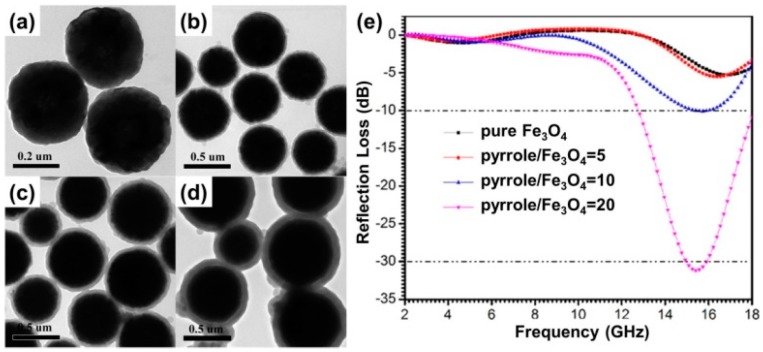
TEM images of (**a**) pure Fe_3_O_4_ microspheres and Fe_3_O_4_@PPy core–shell composite microspheres prepared with different pyrrole/Fe_3_O_4_ ratios: (**b**) 5; (**c**) 10; and (**d**) 20; and (**e**) their microwave reflection losses (absorber thickness = 2.5 mm) in the frequency range of 2–18 GHz. Reprinted with permission from Reference [89].

Moreover, it is worth noting that most composites mentioned above are fabricated from spinel ferrite nanoparticles (ca. 10–100 nm), which usually show very limited permeability in the gigahertz range due to the Snoek’s limit [80], and thus the enhancement of the microwave absorption of these composites is more dependent on the optimized dielectric loss rather than a combination of dielectric and magnetic loss [79]. Inspired by the successful breakthrough of Snoek’s limit in some unique aggregates of spinel ferrite nanoparticles [11,14,81,82,83,84,85]—including urchin-like structure, loose/hollow microsphere, dendrite-like structure, and mesoporous structure—more and more efforts have been devoted to the construction of CP-based composites based on the assembly of spinel ferrite nanoparticles [86,87,88,89]. A two-step oxidative polymerization of aniline monomers was conducted in the presence of Fe_3_O_4_ microspheres with Fe^3+^ and ammonium persulfate (APS) as the oxidants in each step, so that a novel “egg-like” composite of Fe_3_O_4_ microspheres/PANI could be generated (Figure 6) [86]. It was found that embedded Fe_3_O_4_ microspheres would not only modulate the relative complex permittivity but also produce significant magnetic resonance and loss in the composite. More importantly, the composites prepared from the two-step oxidative polymerization using hierarchical magnetic materials had better microwave absorption and environmental stability compared with composites produced by embedding Fe_3_O_4_ nanoparticles, one-step oxidative polymerization, or physical mixture. If the amount of monomers can be precisely controlled or an external field is applied for magnetic collection, various well-defined core–shell composites of Fe_3_O_4_ microspheres and CPs would be obtained [87,88,89]. As shown in Figure 7, the PPy shell thickness can be adjusted from 20 to 80 nm with the variation of the ratio of pyrrole to Fe_3_O_4_ [89]. Investigations of the microwave absorbing properties indicate that the PPy shell plays an important role, and the maximum reflection loss of composite microspheres can reach as much as −31.5 dB (>99.9% absorption) at 15.5 GHz with a matching layer thickness of 2.5 mm. Compared to the physically blended Fe_3_O_4_–PPy composites, Fe_3_O_4_@PPy composite microspheres not only possess better reflection loss performance, but also have a wider absorbing bandwidth of 5.2 GHz (12.8–18.0 GHz) in the Ku band, which is attributed to the intensive synergistic effect of dielectric loss from PPy shells and magnetic loss from Fe_3_O_4_ cores.

In addition to spinel ferrites, the hexagonal ferrites—including M-type, W-type, X-type, Y-type, Z-type, and U-type—are also of great interest as high-frequency MAMs because of their planar magnetic anisotropy and natural resonance in the gigahertz range [90,91,92]. M-type barium ferrites (BaFe_12_O_19_) and strontium ferrites (SrFe_12_O_19_) are two typical examples of the hexagonal group that possess very strong uniaxial anisotropy, high saturation magnetization, and significant permeability [93,94], and thus the integration of BaFe_12_O_19_/SrFe_12_O_19_ and CPs took a considerable share in the field of high-performance MAMs in the past decade [42,95,96,97,98,99,100,101,102]. However, high-temperature sintering (>800 °C) is always necessary for the formation of hexagonal ferrites [103], which results in serious agglomeration of ferrite particles and consequent difficulty in controlling the microstructure of the corresponding composites. This situation limits the development of this kind of MAMs to some extent. Wang et al. reported the synthesis of core–shell BaFe_12_O_19_/PANI nanocomposites with controlled shell thickness by employing BaFe_12_O_19_ nanoparticles derived from sol–gel auto-combustion method as the nucleation sites of PANI [104]. The relatively small particle size more or less improved the homogeneity of BaFe_12_O_19_/PANI nanocomposites, and strong reflection loss over a wide frequency range could be achieved with optimum shell thickness. These results provided a hint that the microwave absorption performance of hexagonal ferrite/CP composites could be further reinforced by reasonable excogitation on the microstructure of the hexagonal ferrite particles. On the other hand, oriented ion substitution in hexagonal ferrites was also confirmed to be a positive method to enhance the microwave absorption performance of hexagonal ferrite/CP composites [105], which might be attributed to the fact that substituted hexagonal ferrites with different magnetic loss and dielectric loss abilities changed the synergetic/complementary behavior between organic and inorganic components [105,106,107]. In view of this, the latest results on the composites of hexagonal ferrites and CPs are mostly constructed with the heteroatoms-substituted hexagonal ferrites [108,109,110,111,112].

### 4.2. Magnetic Metal/CP Composites

**Figure 8:**
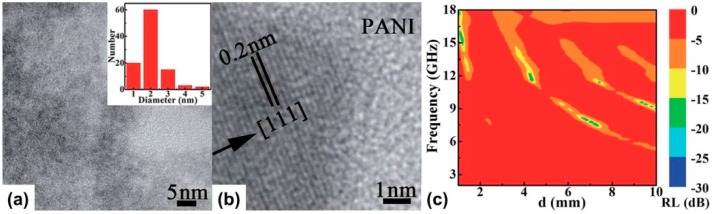
TEM image of (**a**) Ni@PANI nanocomposite and (**b**) HRTEM image of one Ni nanoparticle. The inset in (**a**) shows the size distribution of the Ni nanoparticles; (**c**) Color map of calculated reflection loss values with the thickness of 1.0–10.0 mm as a function of frequency. Reprinted with permission from Reference [118].

Magnetic metal nanoparticles (e.g., Fe, Co, Ni, and their related alloys) generally possess large saturation magnetization, high Snoek’s limit, compatible dielectric loss, and distinguishable permeability in the gigahertz frequency range, which make them better magnetic candidates that can satisfy the design requirements of high-performance MAMs as compared with conventional ferrites [113,114]. However, acidic and oxidative conditions for the polymerization of CPs are unfavorable for magnetic metal nanoparticles, and thus there are only a few accessible papers on the microwave absorption of magnetic metal/CP composites. Dong et al. and Xu et al. prepared Ni/PANI and Ni/PPy composites, respectively, through an in situ chemical oxidative polymerization of monomer in the presence of commercial Ni powder [115,116]. The resultant Ni/PANI and Ni/PPy composites display multiple dielectric relaxations and a natural magnetic resonance. The multiple relaxations for enhanced dielectric loss induced by CPs coatings and proper EM impedance matching due to the synergetic consequence of the Ni cores and polymer shells contribute to the improvement of the EM absorption. Co/PPy composite could be produced with the same method, and its effective microwave absorption was observed in the frequency range of 10.0–18.0 GHz with an absorber thickness of 3.0 mm [117]. More recently, Han et al. embedded superparamagnetic Ni nanoparticles in PANI nanocomposites through a simple oxidation–reduction method followed by an in situ polymerization process [118]. The Ni nanoparticles with single crystalline and single magnetic domain structure possess an average diameter of ~2.1 nm (Figure 8a,b). Such a paramagnetic system with large magnetic moment, fast reaction response to the applied magnetic field, and negligible remanence is beneficial for the formation of antiresonance behavior in permeability. The combined effect of dielectric and magnetic loss is responsible for its excellent microwave absorption, where the optimal reflection loss value of Ni@PANI composites reaches −22.98 dB at 17.8 GHz, corresponding to a very thin absorber thickness of 1.0 mm (Figure 8c).

### 4.3. Transition Metal Oxide/CP Composites

**Figure 9:**
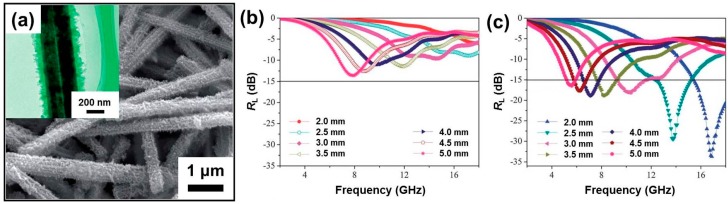
(**a**) SEM image of the branched PANI/α-MoO_3_ nanostructures. The inset shows a TEM image of the branched PANI/α-MoO_3_; The *R*_L_ values of (**b**) the PANI nanorods and (**c**) the branched PANI/α-MoO_3_ nanostructures. Reprinted with permission from Reference [122].

The embedment of magnetic ferrites or metals in CPs indeed brings significant enhancement in microwave absorption, while the employed magnetic components sometimes fail to create sufficient magnetic loss ability as expected [79,104,105,116], which means that the upgraded reflection loss is mainly ascribed to the successful regulation of the relative complex permittivity of CPs in those cases. Compared with magnetic candidates, transition metal oxides have similar influences on the dielectric properties of CPs, and they may possess good dispersion due to the absence of magnetic attraction. As a typical semiconductor, TiO_2_ was reported to be a functional additive that could not only change the charge transport mechanism of PANI, but also increase the dielectric constant [119]. As a result, TiO_2_/PANI composite can generally produce better dielectric loss ability and microwave absorption than pure PANI [120,121]. When uniform α-MoO_3_ nanorods were utilized as the nucleation sites for PANI, small PANI nanorods with average diameter and length of about 55 and 110 nm, respectively, would grow perpendicularly to the α-MoO_3_ surfaces (Figure 9a), and the density of the PANI nanorods could be readily controlled by simply changing the amount of aniline in the reaction system [122]. The branched PANI/α-MoO_3_ heteronanostructures exhibited obviously enhanced microwave absorption in contrast to the pure PANI nanorods (Figure 9b,c), which could be attributed to their improved dielectric loss induced by additional interfacial polarization and their special structural characteristics.

**Figure 10:**
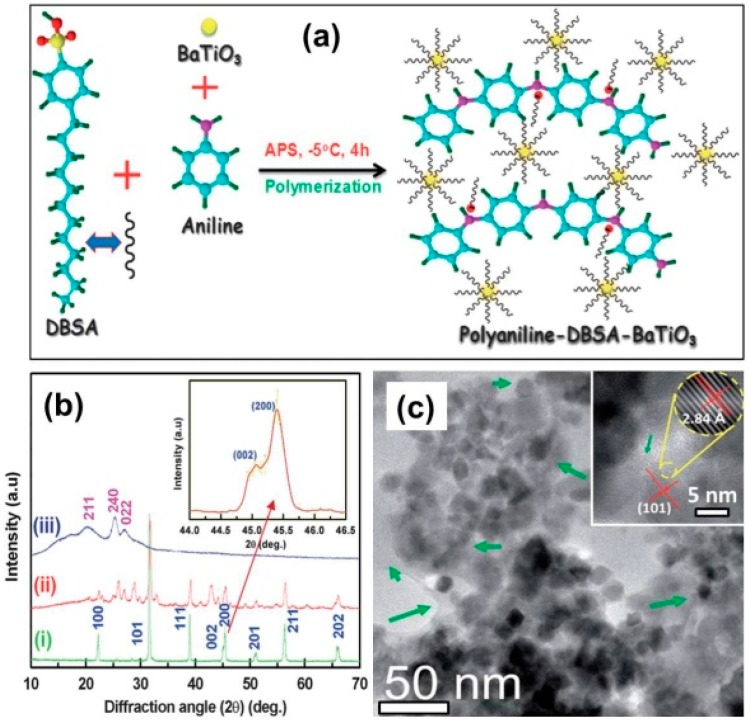
(**a**) Schematic representation on the formation of PANI–BaTiO_3_ nanocomposites by in-situ polymerization; (**b**) XRD patterns of (i) BaTiO_3_, (ii) PANI–BaTiO_3_, and (iii) PANI showing characteristic diffraction planes; TEM image of (**c**) PANI–BaTiO_3_ nanocomposites (green arrows show the presence of PANI); the insect of (**c**) shows inter-planar spacings (red lines) of BaTiO_3_ in PANI-BaTiO_3_ and its enlarged view of lattices (encircled by dashed yellow line) Reprinted with permission from Reference [130]. DBSA: dodecyl benzene sulfonic acid.

Thanks to the universal synergetic effect between CPs and inorganic components, some transition metal oxides with good dielectric property and microwave absorption (e.g., MnO_2_ and BaTiO_3_) were also integrated with CPs to achieve preferable performance [123,124,125,126,127,128,129,130]. For example, Hu et al. obtained γ-MnO_2_/PANI nanocomposites by an in situ polymerization method, and the as-prepared γ-MnO_2_/PANI nanocomposites were found to be a characteristic dielectric medium with multiple loss mechanisms, where the resistance loss from PANI, the dielectric loss from MnO_2_, and polarization relaxation loss from the hybrid interface together contributed to the enhanced microwave absorption [125]. Ting et al. coated BaTiO_3_ particles with PANI shells, and the core–shell composites accounted for stronger and wider reflection loss than naked BaTiO_3_ particles in the frequency range of 2–40 GHz [129]. Saini et al. assembled ultrafine BaTiO_3_ nanoparticles with dodecyl benzene sulfonic acid (DBSA)-doped PANI, and made use of the resultant composite as an EM radiation shield (Figure 10). However, it was revealed that the shielding effectiveness mainly came from the absorption loss rather than reflection principle due to the good dielectric properties of the BaTiO_3_/PANI composite [130].

### 4.4. Carbon/CP Composites

**Figure 11:**
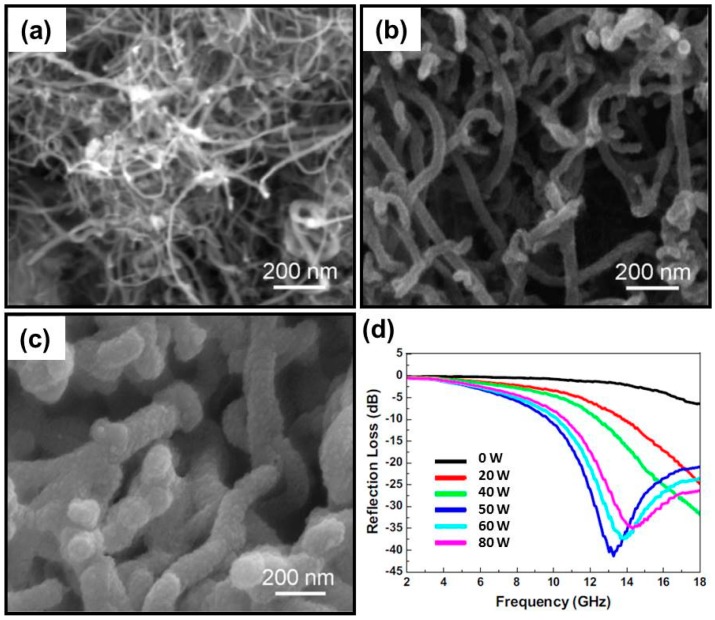
(**a**) SEM image of CNTs; (**b**) SEM image of CNT/PANI hybrids without plasma pretreatment; (**c**) SEM image of CNT/PANI hybrids with plasma pretreatment; (**d**) Frequency dependence of the reflection loss of CNT/PANI hybrids with different plasma pretreatment power (W) in 2–18 GHz. Reprinted with permission from Reference [139].

Carbon materials with diversified forms are a well-known dielectric loss medium and are always the most attractive candidates for microwave absorption due to their characteristic advantages. The integration of carbon materials and CPs may reinforce the dielectric loss properties and provide additional dielectric loss mechanisms (e.g., various polarizations), which will be very promising for strong absorption and wide frequency bandwidth. Furthermore, their low density is also helpful to create a new kind of lightweight MAMs. For example, when carbon black and carbon fiber are coated with a rational amount of PANI, their effective absorption for the incident EM waves will be sufficiently strengthened [131,132]. With the development of carbon science, various fine carbon materials (e.g., carbon nanotubes (CNTs) and graphene) appeared as promising MAMs and became popular carbon additives in CPs [133,134,135,136,137,138,139,140,141,142,143,144,145,146,147]. Sharma et al. found that a PANI–CNTs composite film could provide higher values of dielectric constant and dielectric loss factor than pure PANI film because of the interaction of PANI molecular chains with the surface functionalities of the CNTs [133]. Qiu et al. confirmed that there were apparent resonances between real parts and imaginary parts of relative complex permittivity in multi-walled CNTs (MWCNTs)/PANI composite, leading to the enhanced microwave absorption in the corresponding frequency region [135]. To obtain highly homogeneous carbon/CP composites, several novel methods and techniques were applied to control the polymerization of monomers. Zhang et al. has adopted carboxyl-functionalized MWCNT to enrich pyrrole monomers by acid-base interaction, and the polymerization of pyrrole monomers could be initiated on the surface of multi-walled CNTs rather than in a random way in the solution [138]. It was reported that the electron movement along the wall of CNTs-COOH was blocked in this composite, thus reducing the value of relative complex permittivity and enhancing the microwave absorption as compared with pure PPy and MWCNTs@PPy, and the effective absorption (*R*_L_ < 10.0 dB) could almost cover the whole Ku band with an absorber thickness of 2.0 mm. In addition, plasma-induced graft polymerization—i.e., plasma pretreatment of CNTs and further in situ polymerization of aniline—was also validated to be highly effective for the synthesis of hybrids of CNTs/PANI. As shown in Figure 11a–c, compared with the sample without plasma pretreatment, the one with plasma pretreatment present more uniform PANI coating on the surface of CNTs, because plasma pretreatment made more aniline grafted on the surface of CNTs by oxygen radicals which is beneficial to formation of PANI on the surface of CNTs after polymerization [139]. The conductivity of CNTs/PANI hybrids with plasma pretreatment was higher than that of CNTs/PANI hybrids without plasma pretreatment, and the CNTs/PANI with plasma pretreatment power of 50 W exhibited maximum value of reflection loss (41.37 dB at 13.28 GHz with a thickness of 2.0 mm) (Figure 11d).

**Figure 12:**
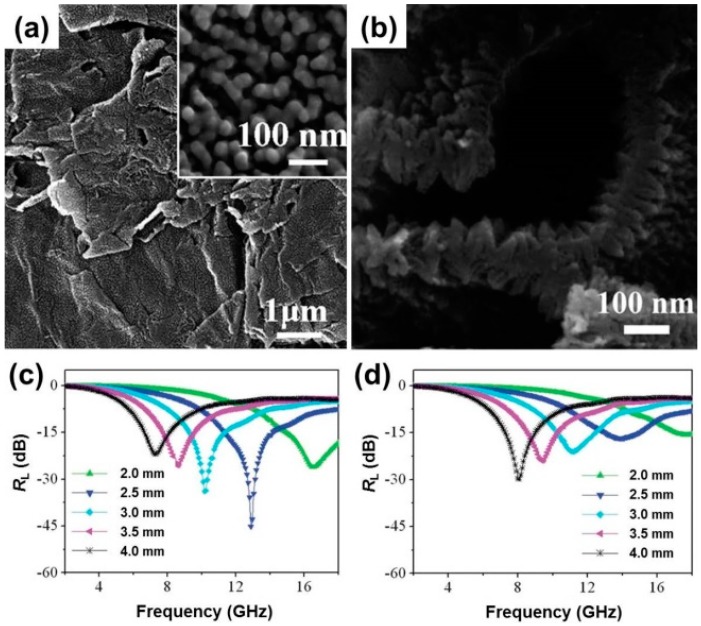
SEM images of graphene/PANI nanorod arrays. (**a**) Top-view, inset: magnified SEM image, and (**b**) the side-view. The reflection loss calculated for (**c**) the graphene/PANI nanorod arrays and (**d**) the PANI nanorods with thicknesses of 2–4 mm. Reprinted with permission from Reference [140].

Compared with CNTs, graphene has better dispersion, and its unique 2D structure is endowed with excellent chemical and physical properties, including high carrier mobility, good conductivity, and large surface area. Graphene/CP composites are now residing at the frontier of functional MAMs, with immense potential for overcoming the challenges related to the performance, functionality, and durability in the field of microwave absorption. Yu et al. pioneered the fabrication of graphene/PANI composite by directing a perpendicular growth of PANI nanorods on the surface of graphene (Figure 12a,b) [140]. The presence of PANI nanorod arrays brought significantly improved microwave absorption properties, where the maximum reflection loss reached −45.1 dB with a thickness of 2.5 mm, and the absorption bandwidth with the reflection loss below −20 dB was up to 10.6 GHz (Figure 12c,d). Experimental results and theoretical simulation indicated that the enhanced EM absorption properties were attributed to the improved dielectric relaxation, the special structural characteristics, and the charge transfer between graphene and PANI nanorods. Duan et al. confirmed that the special π–π interactions between graphene and PANI should be responsible for the dramatically increased microwave absorption, and first-principle calculations also revealed their mutual hybridization and charge transfer [141]. It is worth noting that this interaction is not limited to the system of graphene/PANI, but also works for the composites of graphene/PPy and graphene/PEDOT [145,146,147]. For example, Zhang et al. decorated graphene sheets with PEDOT nanofibres by non-covalent interactions, and the resultant composite showed its maximum *R*_L_ up to −48.1 dB at 10.5 GHz with a thickness of only 2 mm and a very broad absorption bandwidth (5.8–15.8 GHz) over −10 dB by manipulating the thickness from 1.5 to 3.0 mm [145]. Wu et al. yielded a 3D-reduced graphene oxide (RGO)/PEDOT composite by a solution evaporation-induced self-assembly method, and the 3D-RGO/PEDOT composite possessed good microwave absorption capabilities in both low- and high-frequency bands under different thicknesses [146].

### 4.5. Multi-Compound CP-Based Composites

As mentioned above, various binary CP-based composites have made some expected achievements in the field of microwave absorption (especially in the absorption intensity), while they still suffered from a relatively narrow EM response frequency range. In order to further boost performance, more and more interests are focusing on the construction of multi-compound CP composites, where each component should be introduced on purpose to further optimize the EM properties of the final composites. A literature review indicates that there are three main strategies for multi-compound CP-based composites. The first is to introduce a new magnetic component into binary magnetic CPs composites, so that the magnetic loss can be sufficiently reinforced [148,149,150,151]. Yang et al. designed a ternary BaFe_12_O_19_/Y_3_Fe_12_O_12_/PANI composite, and it was found that the combination of hard magnetic ferrites (BaFe_12_O_19_) and soft magnetic ferrites (Y_3_Fe_12_O_12_) not only generated obvious exchange coupling behavior and magnetic loss ability, but also brought typical dielectric resonances [148]. As a result, this ternary BaFe_12_O_19_/Y_3_Fe_12_O_12_/PANI composite showed much better microwave absorption than binary BaFe_12_O_19_/PANI composite. Although the exchange coupling behavior would disappear when the garnet-type ferrites (Y_3_Fe_12_O_12_) were replaced by various spinel ferrites (e.g., CoFe_2_O_4_, MnFe_2_O_4_, Ni_0.8_Zn_0.2_Fe_2_O_4_), the ternary composites could still consume more incident EM waves [149,150].

**Figure 13:**
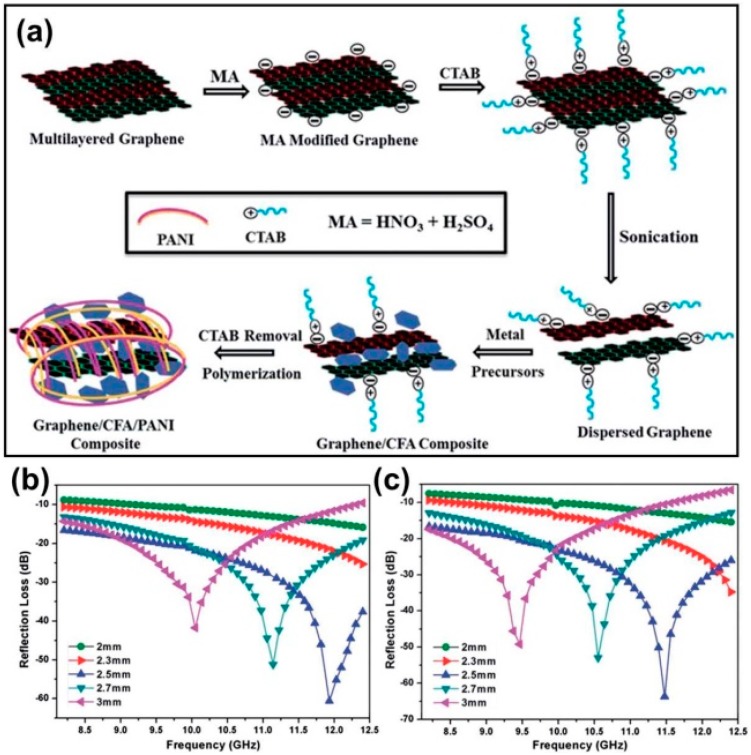
(**a**) Schematic representation of the preparation of the nanocomposites, CTAB: cetyltrimethylammonium bromide; (**b**) Reflection loss vs. frequency plots of graphene/CFA (CuFe_10_Al_2_O_19_) and (**c**) graphene/CFA/PANI. Reprinted with permission from Reference [160].

In the second way, there are two dielectric components and one magnetic component in the multi-compound composites. Due to the high density of magnetic ferrites/metals, the improvements in EM properties by constantly accumulating magnetic components will be contrary to the requirements of practical applications, and thus employment of another dielectric medium becomes a more prospective mode that can maintain the specific gravity of the composites within the acceptable level. Generally, this kind of multi-compound CP-based composite is constructed on the basis of a simple magnetic composite, and the CPs will act as a shell layer and organic binder [152,153,154,155,156,157,158,159,160,161,162,163]. For example, Han et al. prepared FeNi@C@PANI nanocomposite by combining the arc-discharge process and an in situ chemical polymerization reaction. It was found that the introduction of PANI could enhance the relative complex permittivity to some extent, and pull down the density of the composite effectively. Compared with FeNi@C, the FeNi@C@PANI nanocomposite could perform similar microwave absorption with thinner absorber thickness [152]. Yang et al. also confirmed that a PPy-encapsulated nickel-coated graphite nanosheet had much better microwave absorption properties than a binary nickel-coated graphite nanosheet and a PPy-coated graphite nanosheet [155]. Bhattacharya et al. designed a ternary composite by wrapping CuFe_10_Al_2_O_19_ (CFA)-decorated graphene with PANI (as shown in Figure 13), and the resultant graphene/CFA/PANI composite could always create more powerful absorption than graphene/CFA in X band with different absorber thicknesses. The excellent microwave absorption of the prepared nanocomposites was due to the summation of good impedance matching characteristics, good complementarity of dielectric loss (due to graphene and PANI) and magnetic loss (due to CFA), good electrical conductivity, special structural characteristics, etc. [160]. In addition to various carbon substrates, BaTiO_3_ and TiO_2_ can be also utilized as the subprime dielectric medium to regulate the EM properties of ternary CP-based composites [67,163].

**Figure 14:**
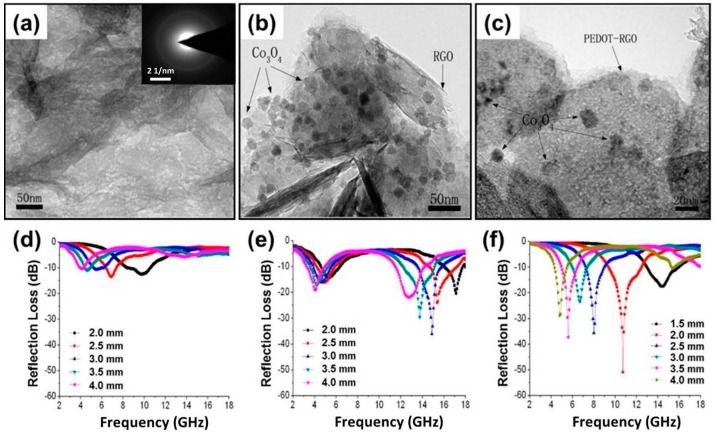
TEM image of (**a**) PEDOT–RGO (reduced graphene oxide); (**b**) RGO–Co_3_O_4_; and (**c**) PEDOT-RGO-Co_3_O_4_; Reflection loss curves of (**d**) PEDOT–RGO; (**e**) RGO–Co_3_O_4_; and (**f**) PEDOT–RGO–Co_3_O_4_. Reprinted with permission from Reference [166].

Decorating pre-prepared carbon/CPs with magnetic nanoparticles is another alternative route to multi-compound composites with binary dielectric media [164,165,166,167,168,169,170,171,172]. Chen et al. and Zhang et al. deposited CoFe_2_O_4_ and Fe_3_O_4_ nanoparticles on the surfaces of expanded graphite/PANI and CNTs/PANI, respectively, and the advanced binary dielectric system and the synergetic magnetic loss were considered to be responsible for the enhanced microwave absorption [164,165]. Huang’s group fabricated a series of ternary MAMs by decorating graphene/CPs with various spinel ferrite nanoparticles [166,167,168,169,170,171,172]. As shown in Figure 14, well-dispersed Co_3_O_4_ nanoparticles were located at the surface of PEDOT/graphene (PEDOT-GN), and the microwave absorption results validated that the ternary PEDOT–RGO–Co_3_O_4_ composite could produce better reflection loss characteristics than binary PEDOT–RGO and RGO–Co_3_O_4_ composites [166]. A similar phenomenon could be also found in analogous composites; e.g., graphene/PANI/NiFe_2_O_4_, graphene/PEDOT/NiFe_2_O_4_, grapheme/PEDOT/CoFe_2_O_4_, graphene/PANI/Fe_3_O_4_, graphene/PPy/Fe_3_O_4_, graphene/PANI/FeNi_3_ [167,168,169,170,171,172].

As mentioned above, well-matched characteristic impedance and good reflection loss property can be also achieved by rational manipulation of absolute dielectric loss systems [120,121,122,123,124,125,126,127,128,129,130,131,132,133,134,135,136,137,138,139,140,141,142,143,144,145,146,147], and thus the third strategy for multi-compound CP-based composites is derived from the combination of various dielectric components. For instance, Sambyal et al. blended PANI, barium strontium titanate, and expanded graphite into ternary composites with different weight ratios, and they found that the weight ratio could impact the microwave absorption of these composites greatly [173]. When the weight ratio of PANI to barium strontium titanate to expanded graphite was 1:0.1:2, the corresponding composite, PBE112, would display excellent reflection loss properties (ca. −70 dB) in the whole Ku-band. Ni et al. assembled a one-dimensional CNT@BaTiO_3_@PANI multiphase heterostructure composite via coupled sol–gel method and in situ polymerization [174]. The CNT@BaTiO_3_@PANI composite gave very broad response frequency bandwidth—less than −10 dB over 7.9–15.0 GHz and less than −20 dB over 10.0–15.0 GHz—with a thickness of 3.0 mm. The excellent microwave absorption property was believed to be linked to its special structural characteristics, well-matched characteristic impedance, and interfacial polarization induced by multiple interfaces in the composite.

**Figure 15:**
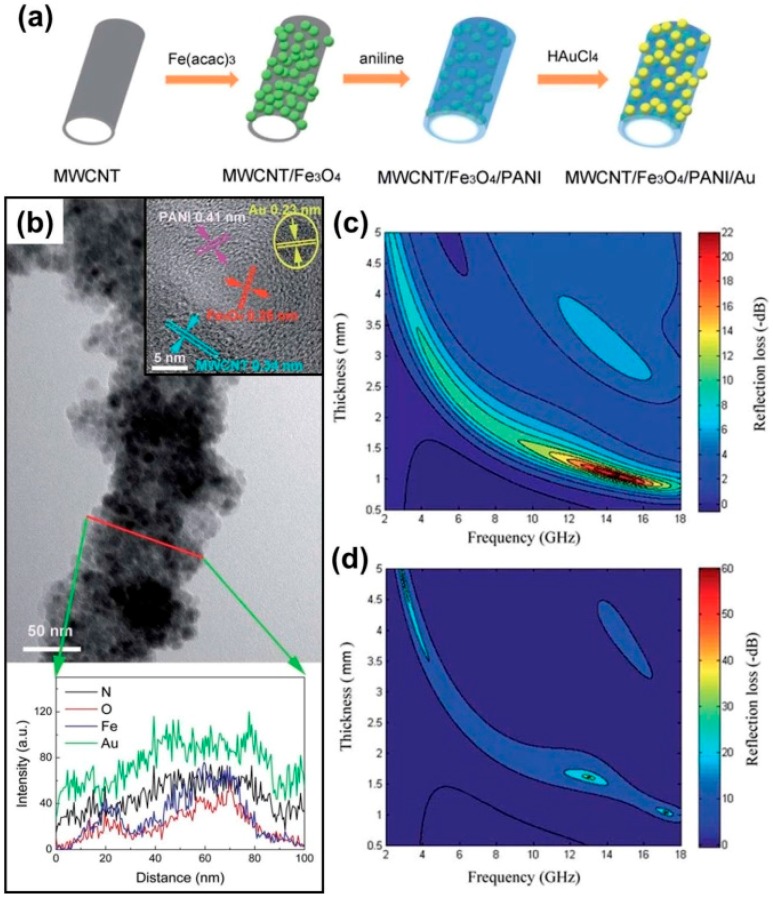
(**a**) Schematic illustration of the fabrication of MWCNT/Fe_3_O_4_/PANI/Au hybrid nanotubes. (**b**) TEM images of hybrid nanotubes; the bottom image shows the EDS (Energy-dispersive X-ray Spectrometer) line scan profiles of N, O, Fe, and Au; the top inset shows the HRTEM image, the yellow circle labels the gold nanoparticles. Microwave absorption characteristics of (**c**) ternary MWCNT/Fe_3_O_4_/PANI hybrid nanotubes and (**d**) quaternary MWCNT/Fe_3_O_4_/PANI/Au hybrid nanotubes. Reprinted with permission from Reference [175].

More recently, several groups started to construct quaternary composites as high-performance MAMs. As shown in Figure 15, Liu et al. dispersed Au nanoparticles on the external surface of core–multishell MWCNT/Fe_3_O_4_/PANI hybrid nanotubes, and Au nanoparticles therein could improve the impedance matching and function as a reflector, which induced more EM waves to be transmitted into the composite and promoted multiple reflection behaviors inside the composites [175]. As a result, the quaternary MWCNT/Fe_3_O_4_/PANI/Au hybrid composite produced better microwave absorption than the ternary MWCNT/Fe_3_O_4_/PANI hybrid composite. In addition, quaternary graphene@Fe_3_O_4_@SiO_2_@PANI and FeNi_3_@SiO_2_@rGO/PANI composites were also reported [176,177]. Although strong absorption and wide response frequency range could be realized in these complicated composites, the specific loss mechanisms have not been explained clearly. Especially, the effects of wave-transparent SiO_2_ were not mentioned.

### 4.6. Multi-Layer CP-Based Composites

In the practical applications of various MAMs, they are generally filled into some polymer matrixes to form a microwave-absorbing coating. Some theoretical analyses have predicted that optimization of the microwave absorbing coating—including the construction of multi-layer geometrical configuration and manipulating microstructure/morphology of MAMs—could work for the broadened absorption bandwidth [178,179,180], and this has been firmly supported by a series of experimental results [181,182,183,184,185]. Inspired by these impressive advantages, multi-layer CP-based MAMs have also been developed to pursue better performance. For example, Xu et al. designed double-layer MAMs with PANI as the absorbing layer and PANI/Fe_3_O_4_ composite as the matching layer. When the thicknesses of the absorbing layer and the matching layer were 0.4 and 0.6 mm, the maximum *R*_L_ would reach up to −42 dB at 29.27 GHz, and the absorption bandwidth with *R*_L_ below −10 dB was about 11.8 GHz [186]. Egami et al. reinforced the microwave absorption properties of a PPy-coated non-woven fabric sheet by accumulating its stacked numbers, and they found a stack of ten sheets could consume over 95% of incident EM waves in the range of 75–110 GHz [187]. Micheli et al. synthesized multilayered MAMs with rational allocation to layer stacking of graphene nanoplatelets, carbon nanofibers, MWCNTs, and PANI, and their simulated results were consistent with the measured data, which confirmed that this heterogeneous multilayer structure was beneficial for microwave absorption [188].

## 5. Comparison of Different CP-Based MAMs

As presented above, there are many kinds of CP-based composites that can display considerable microwave absorption properties. In Table 1, we summarize the reflection loss characteristics of some representative CP-based composites. However, one can see that these composites always perform different matched thickness and absorption frequency, and thus it is very hard to find an assessment system that can feature them comprehensively. If the assessment only focuses on maximum *R*_L_ values and effective response bandwidth, multi-compound and multi-layer CP-based composites will be preferable candidates to a certain degree, which may point to the direction of the future development of CP-based MAMs.

## 6. Conclusions and Outlook

This review highlighted the recent developments related to conjugated polymer-based composites as promising microwave absorbing materials (MAMs). Some studies reported that rational design on the microstructure of conjugated polymers (CPs) was an effective strategy to regulate their EM properties and improve the matching of characteristic impedance, and as a result, some CPs with unique microstructures—such as PANI microrods, PANI nanoparticles, multi-shelled PEDOT hollow microspheres, and highly uniform core–shell PPy@PANI—displayed significantly enhanced microwave absorption as compared with their conventional individual components. There have also been more tremendous interests in constructing heterogeneous CP-based composites with various magnetic ferrites, magnetic metal, transition metal oxides, and diversified carbon materials for microwave absorption application. The integration of CPs and these inorganic additives could generally create obvious synergetic effects and complementary behaviors, as well as more loss mechanisms (e.g., various polarization relaxations), which contribute to the microwave absorption performance greatly. To further upgrade the attenuation for incident EM waves, the EM properties of CP-based composites could be optimized by incorporating secondary magnetic components or dielectric components. Therefore, ternary and quaternary CP-based composites are making breakthroughs and are becoming a popular approach for high-performance MAMs. In addition, CP-based composites also share their applications in multi-layer MAMs, which were more suitable for practical microwave absorption. The rational arrangement in multi-layer MAMs made great contribution to broadened response bandwidth.

Although significant progress has been made in these CP-based composites, a gap to real industrial applications still exists, as current composites cannot fulfill the particular demands for MAMs, which means that more efforts are still required in this field. First, the frequency range upon the effective absorption is still less than the expected bandwidth, so that accumulating the absorber thicknesses or constructing multi-layer configuration is extensively adopted to broaden the response frequency range in most research cases. As mentioned above, rational design on the microstructure may reinforce the microwave absorption of pure CPs, while this motivation is rarely referred for CP-based composites. Constructing multi-compound composites with unique microstructure (e.g., hollow, yolk–shell, smaller particle size)—which can intensify the reflection and scattering behaviors of EM waves inside MAMs—will be a promising and challenging task to produce desirable microwave absorption performance. Second, many studies intently pursue the enhancement of microwave absorption regardless of other assessment criteria. Actually, low density and loading amount are very important in practical applications, especially in the field of aeronautics and astronautics. Therefore, with the premise of comparable functionality, the employment of ultra-light additives is quite meaningful for various CP-based composites. Third, some CPs and magnetic components usually suffer from some rigorous conditions and long-time application, and thus the stability and lifetime of CP-based composites should also be worth considering. In summary, CP-based composites exhibit their potential as a promising kind of MAMs, although they are at an initial stage from the related research. It is believed that novel CP-based composites with ideal compositions and optimal microstructures will present a bright future in the field of microwave absorption.

## Figures and Tables

**Table 1:** Performance comparison of representative CP-based MAMs.

Entry	Absorbers	Thickness (mm)	Maximum *R*_L_ and Frequency	Bandwidth over −10 dB (Range, GHz)	Reference
1	3D PPy aerogel	3.0	−22.5 dB at 12.0 GHz	5.0 (10.0–15.0)	[60]
2	PANI nanoparticle	2.0	−18.8 dB at 17.2 GHz	3.9 (14.1–18.0)	[62]
3	Fe_3_O_4_ nanoparticle/PPy	1.7	−35.1 dB at 16.7 GHz	2.1 (15.9–18.0)	[79]
4	Fe_3_O_4_ microspheres/PANI	3.0	−31.3 dB at 9 GHz	2.2 (7.6–9.8)	[86]
5	BaFe_12_O_19_/PANI	2.0	−20.0 dB at 14.5 GHz	4.0 (12.8–16.8)	[95]
6	Ni/PANI	1.0	−23.0 dB at 17.8 GHz	2.5 (15.5–18.0)	[118]
7	α-MoO_3_/PANI	2.0	−34.0 dB at 16.8 GHz	2.8 (15.2–18.0)	[122]
8	MCNT-COOH/PPy	3.5	−16.0 dB at 11.5 GHz	4.5 (9.5–14.0)	[138]
9	Graphene/PANI	2.5	−45.1 dB at 12.9 GHz	5.4 (10.6–16.0)	[140]
10	0.9BaFe_12_O_19_/0.1Y_3_Fe_5_O_12_/PANI	2.9	−40.8 dB at 9.9 GHz	5.5 (6.8–12.3)	[148]
11	Graphene/CuFe_10_A_l2_O_19_/PANI	2.5	−63.6 dB at 11.5 GHz	4.5 (8.0–12.5)	[160]
12	NiFe_2_O_4_/graphene/PEDOT	2.5	−50.5 dB at 12.5 GHz	5.3 (11.0–16.3)	[168]
13	Graphene/Fe_3_O_4_/SiO_2_/PANI	2.5	−40.7 dB at 12.5 GHz	5.8 (10.5–16.3)	[176]
14	Fe_3_O_4_-PANI layer/PANI layer	1.0	−42.0 dB at 29.27 GHz	11.8 (25.5–37.3)	[186]
